# Supplementary material for: Effects of expanding imports on urban manufacturing employment: Evidence from China
Source: PLoS One. 2024 Jan 29;19(1):e0296961. doi: 10.1371/journal.pone.0296961 (PMC10824462; doi:10.1371/journal.pone.0296961)
Supplement: Attachment — Submitted filename: Response to reviewers.docx [file pone.0296961.s004.docx]

Dear Editor,

Thank you for your revision recommendations. We have made the following revisions according to the recommendations and requirements.

**Recommendation 1: Writing style**

Ensure that your writing is clear, concise, and free of jargon. Aim to use straightforward language and sentence structures that will be easily understood by readers from various backgrounds.

**Reply:** We have resubmitted our paper to the language polishing agency (American Journal Experts, AJE) which collaborates with our university, they have polished and modified the language, the editor has re-edited concisely to avoid wordiness, help avoid jargon, and pay special attention to sentence structure.

**Recommendation 2: Paper structure**

Review the overall structure of your paper, making sure that each section flows logically and cohesively. Use headings and subheadings to guide the reader through your argument and to highlight key points.

**Reply:** The structure of the paper has been fine-tuned, with benchmark estimation, robustness test, mechanism test, and heterogeneity analysis included in the empirical research section. The chapters of the paper have been adjusted from the original 9 chapters to 7 chapters. Except for the first chapter, each other chapter uses headings and subheadings to guide the readers through our arguments and highlight key points.

**Recommendation 3: Introduction and conclusion**

Revise your introduction to more effectively present the research question, its significance, and your main findings. Similarly, refine your conclusion to provide a clear summary of your findings and their implications for policy and future research.

**Reply:** We have included the research questions, significance and main findings in the introduction part. Summary of findings, implications for policy and future research are also included in the conclusion part.

**Recommendation 4: Figures and tables**

Ensure that all figures and tables are clearly labeled, easy to read, and directly relevant to your research question. Include concise captions that explain the content and significance of each figure or table.

**Reply:** We have labeled the figures and tables clearly, and included concise caption to explain the content and significance of each figure and table.

**In addition, the academic editor put forward some additional requirements, we response as follows.**

**Requirement 1:** Please ensure that your manuscript meets PLOS ONE's style requirements, including those for file naming.

**Reply:** We have strictly typeset the manuscript according to the PLOS ONE's style requirements, including those for file naming.

**Requirement 2:** Please note that PLOS ONE has specific guidelines on code sharing for submissions in which author-generated code underpins the findings in the manuscript. In these cases, all author-generated code must be made available without restrictions upon publication of the work and ensure that your code is shared in a way that follows best practice and facilitates reproducibility and reuse.

**Reply:** We have submitted the data and code to Dryad. DOI: 10.5061/dryad.w6m905qvr.

**Requirement 3:** Funding statement

**Reply:** In our updated Funding Statement, we have amended the statement that declares that there is no additional external funding received for this study, the statement has also been included in the cover letter.

**Requirement 4:** Data set and figure files

**Reply:** We have submitted the source and description of data set in the uploaded Supporting Information files. The data set have been uploaded to Dryad, DOI: 10.5061/dryad.w6m905qvr.

The figures and tables have been transferred into TIFF format and included in the Supporting Information files.

The revised manuscript has been submitted, we look forward to receiving your reply.

Yours Sincerely,

Chunyan Zhao, Ass. Prof. PhD.
